# Integrative Analysis Identifies Cell-Type-Specific Genes Within Tumor Microenvironment as Prognostic Indicators in Hepatocellular Carcinoma

**DOI:** 10.3389/fonc.2022.878923

**Published:** 2022-05-30

**Authors:** Zi-Li Huang, Bin Xu, Ting-Ting Li, Yong-Hua Xu, Xin-Yu Huang, Xiu-Yan Huang

**Affiliations:** ^1^ Department of General Surgery, Shanghai Jiaotong University Affiliated Sixth People’s Hospital, Shanghai, China; ^2^ Department of Radiology, Xuhui District Central Hospital of Zhongshan Hospital, Fudan University, Shanghai, China; ^3^ Department of General Surgery, The Tenth People’s Hospital of Tongji University, Shanghai, China; ^4^ Department of Infectious Disease, Shanghai Jiao Tong University Affiliated Sixth People’s Hospital, Shanghai, China

**Keywords:** recurrence-free survival, single-cell RNA sequencing, tumor microenvironment, T cell, immune response

## Abstract

**Background:**

Hepatocellular carcinoma (HCC) is a leading cause of cancer-related mortality worldwide, but effective early detection and prognostication methods are lacking.

**Methods:**

The Cox regression model was built to stratify the HCC patients. The single-cell RNA sequencing data analysis and gene set enrichment analysis were employed to investigate the biological function of identified markers. PLCB1 gain- or loss-of-function experiments were performed, and obtained HCC samples were analyzed using quantitative real-time PCR and immunohistochemistry assay to validate the biological function of identified markers.

**Results:**

In this study, we developed a model using optimized markers for HCC recurrence prediction. Specifically, we screened out 8 genes through a series of data analyses, and built a multivariable Cox model based on their expression. The risk stratifications using the Eight-Gene Cox (EGC) model were closely associated with the recurrence-free survivals (RFS) in both training and three validation cohorts. We further demonstrated that this risk stratification could serve as an independent predictor in predicting HCC recurrence, and that the EGC model could outperform other models. Moreover, we also investigated the cell-type-specific expression patterns of the eight recurrence-related genes in tumor microenvironment using single-cell RNA sequencing data, and interpreted their functional roles from correlation and gene set enrichment analyses, *in vitro* and *in vivo* experiments. Particularly, PLCB1 and SLC22A7 were predominantly expressed in malignant cells, and they were predicted to promote angiogenesis and to help maintain normal metabolism in liver, respectively. In contrast, both FASLG and IL2RB were specifically expressed in T cells, and were highly correlated with T cell marker genes, suggesting that these two genes might assist in maintaining normal function of T cell-mediated immune response in tumor tissues.

**Conclusion:**

In conclusion, the EGC model and eight identified marker genes could not only facilitate the accurate prediction of HCC recurrence, but also improve our understanding of the mechanisms behind HCC recurrence.

## Introduction

Hepatocellular carcinoma (HCC) is one of the most common malignancies and a leading cause of cancer-related mortality worldwide (1). Common etiologic factors for HCC are HBV/HCV infection, use of alcohol and tobacco, and cirrhosis (2). As few clinical signs can be observed in early-stage HCC, delayed diagnoses often lead to poor prognoses (3). Although targeted therapy and immunotherapy have improved the prognoses of HCC patients to some extent, the odds of recurrence within five years are still relatively high (4–6).

Targeted therapies and immunotherapies are considered promising strategies for unresectable HCC, and therapeutic breakthrough now heavily relies on the identification of potential molecular markers (7). So far, there are several models for predicting HCC prognosis, using different gene signatures such as HOXD9, SPP1, SPINK1, TXNRD1 and MAGEB6, and the satisfying performances of those models suggested that these genes could serve as possible drug targets (8, 9). Since inflammation and immune response play an essential role in the initiation and development of HCC, molecules participating in these processes are receiving growing attention (10). High density of CD3+ and CD8+ cells, along with PD-L1 expression are considered as useful markers for post-surgical, relapse-free survival (1). The α-fetoprotein (AFP) levels are found to be an indicator of recurrence after direct-acting antiviral agent (DAA) therapy for HCC patients with hepatitis C virus infection (11, 12), while another study has demonstrated that IL-11 is associated with HCC recurrence in patients after surgical resection, and blocking IL-11-STAT3 signaling could help prevent post-surgical recurrence (13). Aside from these findings, there is a study focused on predictive lncRNA markers in HCC, and MSC-AS1, POLR2J4, EIF3J-AS1, SERHL, RMST, and PVT1 were recognized as prognostic indicators, which are mainly associated with TGF-β signaling and cellular apoptosis. Meanwhile, a recent study has investigated DNA methylation-driven genes in the growth and metastasis of HCC, concluding that SPP1 and LCAT are candidate markers for HCC recurrence (14). With the aid of advanced sequencing technologies, it is now possible to explore mechanisms behind HCC tumorigenesis from diverse perspectives. Nonetheless, novel predictive biomarkers of HCC recurrence are still inadequately addressed. Here we attempt to develop a predictive model using optimized markers for postsurgical recurrence of HCC, and to interpret their potential functional significance in HCC recurrence.

## Materials and Methods

### Data Download and Preprocessing

The three pre-normalized gene expression datasets with clinical characteristics were downloaded from the Gene Expression Omnibus (GEO) database under accession number GSE14520 (15) and GSE76427 (16). The Clinical Proteomic Tumor Analysis Consortium (CPTAC) HCC data was obtained from NODE (The National Omics Data Encyclopedia) with under accession number OEP000321. The clinical characteristics of the three cohorts were summarized in **Supplementary Table S1**. Gene expressions were logarithmically transformed and then used for downstream data analyses.

### Survival Analysis

The Cox proportional hazard regression analysis was employed to identify the recurrence-related genes, and a multivariable Cox model was constructed using identified markers. Specifically, the differential expression analysis was first conducted to identify differentially expressed genes (DEGs) in HCC (Student *t* test, *P*-value < 0.05). Subsequently, the univariable Cox proportional hazard regression analysis was performed to detect DEGs correlated with recurrence-free survival (RFS) in HCC patients (Log rank test, *P*-value < 0.05). It should be noted that for each sample, the expression status of any given gene was denoted as high or low, using its median expression levels in all samples as a cutoff. Furthermore, the MMPC (17) (Max-Min Parents and Children) algorithm was used to identify the best combination of gene signatures with a *P*-value lower than 0.05, and the multivariable Cox model was built based on the selected gene set. The HCC patients were stratified into two risk groups according to the median value of the risk scores, which were estimated using the Cox model and the expression status of the eight signature genes. The Cox model and MMPC algorithm were implemented in R survival (18) and MXM packages (19).

### The Comparison of Cox Models Built on Different Signatures

The hazard ratios and 95% confidence intervals were calculated using different Cox regression models. The forest plots were plotted using R survcomp (20).

### Single-Cell RNA Sequencing Data

We collected the HCC single-cell RNA sequencing (scRNA-seq) data from GEO database under accession number GSE125449 (21), from which the Set 1 was used in this study. The cell types were pre-defined in the previous study. The t-Distributed Stochastic Neighbor Embedding (t-SNE) analysis and dimensionality reduction were conducted to visualize the clustering of cell types. The cell-type-specific marker genes were identified from the differential expression analysis. The scRNA-seq data analysis was implemented in R Seurat package (22).

### Gene Set Enrichment Analysis (GSEA)

Prior to GSEA, the Spearman correlation analysis was first conducted in this study. Subsequently, genes were ranked according to Spearman correlation coefficients (SCC). GSEA was then conducted to identify the gene sets enriched by genes correlated with identified markers. This analysis was implemented in R clusterProfiler (23). The hepatocyte specific markers were obtained from a liver single-cell RNA-seq study (24).

### Kyoto Encyclopedia of Genes and Genomes (KEGG) Pathway Analysis

The core genes in the set of T cell marker genes were subjected to the KEGG pathway analysis. The Fisher’s exact test was used to measure the statistical significance. The KEGG pathway analysis was implemented in R clusterProfiler package (23).

### Cell Culture

The HCC cell lines, MHCC97H, MHCC97L, SMMC7721 and Hep3B, were cultured following procedures stated in a previous report (25). Specifically, a highly metastatic HCC cell line, MHCC97, were originated from LCI-D20 tumor, which was a subclone possessing high metastatic potentials (26) (up to 100% pulmonary metastatic rate in MHCC97H was reported using orthotopic inoculation). The human HCC cell line Hep3B with very low invasive potentials (27) was also prepared in this study. Human HCC cell lines Hep3B and MHCC97H were obtained from the Liver Cancer Institute, Shanghai Medical College of Fudan University, and cultured in Dulbecco modified Eagle medium (DMEM) (Gibco-BRL, Gaithersburg, MD, USA) supplemented with 10% fetal bovine serum (HyClone, Logan, UT, USA) in a humidified incubator containing 5% CO_2_ at 37°C. In all experiments, no antibiotics were used.

### Overexpression and Knockdown of PLCB1 Gene

The PLCB1 cDNA was cloned following a previous study (28), and a scrambled sequence was used as a control. The small interfering RNA (siRNA) sequences were designed to target PLCB1. Cell transfection was performed using the Lipofectamine 2000 reagent (Thermo Fisher Scientific) in antibiotic-free medium. The detailed siRNA sequences were as follows:

(+) 5’-CAGAAGAGUGUCAGAACAATT-3’,

(–) 5’-UUGUUCUGACACUCUUCUGTT-3’.

### Clinical Specimens

A total of 32 snap-frozen HCC tissues were obtained from the Hospital Clinic and examined using quantitative real-time PCR (qPCR). Informed consent was obtained from each patient, and the Research Ethics Committee of Hospital approved all aspects of this study. Cases with hepatitis B from 2019 to 2021 were confirmed by experienced pathologists based on WHO criteria. The clinical characteristics of the three cohorts were summarized in **Supplementary Table S1**.

### Quantitative Real-Time PCR

Quantitative PCR assays of cDNA were performed using a CFX96 Real-time PCR system (Bio-Rad). Relative expressions of the target transcripts were quantified and normalized to the expression of the reference gene GAPDH. Specifically, expressions of target genes in each sample were first assessed by qRT-PCR independently. Target cDNAs were amplified using the following probe set:

B3GAT3: forward 5’-CTCGGCCAGCCATGTGAC-3’; reverse 5’-TCCAGCCATATCCACAGGGA-3’.

EEF1D: forward 5’-TTCATCAGTCTTCCCGCGTC-3’; reverse 5’-CTTGTTGACCCAGATCCCCC-3’.

NRP1: forward 5’-GACCTGGGGGAGGAGAAGAT-3’; reverse 5’-GATCCTGAATGGGTCCCGTC-3’.

PLCB1: forward 5’-GGACTGACCCTCAGGGATTTT-3’; reverse 5’-AAGCCACGAGATTCAAATGGG-3’.

FASLG: forward 5’-CTTGGTAGGATTGGGCCTGG-3’; reverse 5’-CTGGCTGGTAGACTCTCGGA-3’.

IL2RB: forward 5’-CATGTCTCAGCCAGGGCTTC-3’; reverse 5’-GTTGCATCTGTGGGTCTCCA-3’.

SLC22A7: forward 5’-CCAGAGTCCAAGGGTCTATGT-3’; reverse 5’-ATCAAGGATGGATGAGCAGAG-3’.

STX11: forward 5’-ACACGTAAGCAGGAAGCAGC-3’; reverse 5’-GTGCAGTGGGCCAAATGATG-3’.

GAPDH: forward 5’-ACCCACTCCTCCACCTTTG-3’; reverse 5’-CTCTTGTGCTCTTGCTGGG-3’.

### Immunohistochemistry Assay

Intratumoral microvessels immunostained for CD31 were counted using the method described by Weidner et al. (29). All slides were independently assessed by two board-certified pathologists that were blinded in this experiment. Any disagreements in the microvessel count were resolved by consensus.

### Cell Invasion Assay

Cell invasion assays were performed in a Transwell apparatus (Millipore), following the manufacturer’s instructions. Briefly, after being serum-starved for 24 h, the cells were seeded in the top chamber of the Transwell apparatus (100 μL DMEM, 5 × 104 cells/well), which was coated with collagen IV, and 600 μL conditioned medium was added to the bottom chamber of the apparatus. The cells were subsequently incubated for 48 h at 37°C and then fixed with 0.1% methanol for 10 min before hematoxylin staining. Migrated cells were counted in three independent experiments, and the results were presented as means ± SD.

### MMP9 Activity Assay

MMP9 activity was measured using a human MMP9 Activity ELISA System (Amersham Pharmacia Biotech, Piscataway, NJ), following the manufacturer’s instructions. The plate was read at 450 nm in a SPECTRAmax 250 Microplate Spectrophotometer (Molecular Devices, Sunnyvale, CA). Assays were repeated in triplicate.

### *In Vivo* Study

Male athymic BALB/c nu/nu mice of 18–20 g at 5 weeks’ age were handled according to the recommendations of the National Institutes of Health Guidelines for Care and Use of Laboratory Animals. The experimental protocol was approved by the Shanghai Medical Experimental Animal Care Committee. Human HCC tumor models produced by MHCC97H were established in nude mice by orthotopic inoculation. We silenced the expression of PLCB1 in MHCC97H cells using the small interface RNA (siRNA). Then, 1 × 10^5^ cells were subcutaneously injected to form the tumors, which were inoculated *in situ* in nude mice (KD group), while CON group presented siRNA negative group. Briefly, the left lobe of the liver was exposed under anesthesia, and a part of the liver surface was mechanically injured with scissors. Then, a piece of MHCC97H tumor tissue (size 2 × 2 × 2 mm) was fixed within the liver tissue.

## Results

### Identification of Recurrence-Related Genes in HCCs

**Figure 1:**
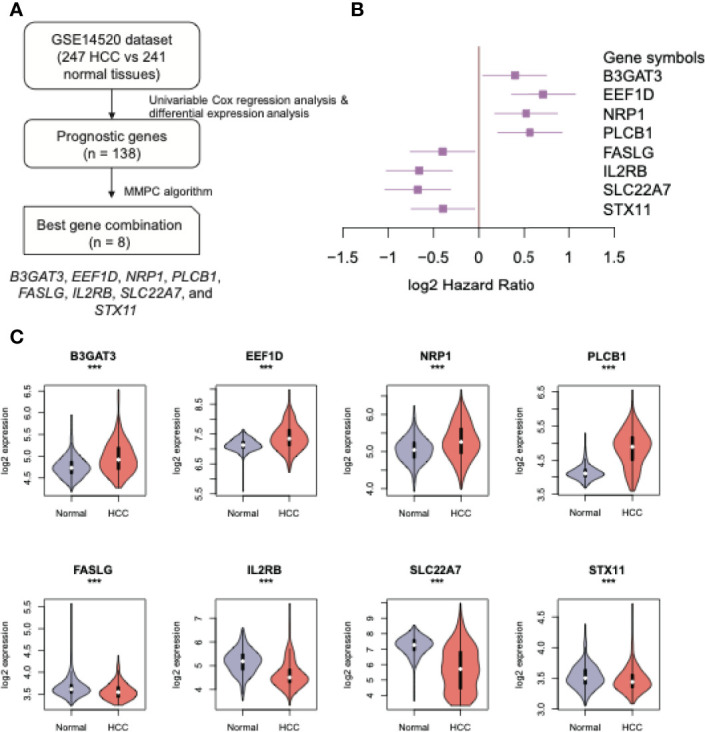
The eight recurrence-related genes in HCC. **(A)** The workflow for the identification of the eight recurrence-related genes. **(B)** The forest plot displays the log2 (hazard ratios) (the boxes) and its 95% confidence intervals (the two ends) of the eight recurrence-related genes. **(C)** The expression patterns of the eight recurrence-related genes in HCC and adjacent normal tissues. The orange and purple colors represent the HCC and adjacent normal tissues, respectively. ***: P-value < 0.05.

To build a predictive model for HCC recurrence, we first conducted univariable Cox regression analyses and a differential gene expression analysis to identify recurrence-related genes, using a gene expression dataset with a long-term follow-up of patients from GEO under accession number GSE14520 (**Figure 1A**). Specifically, we identified 138 recurrence-related genes (**Supplementary Table S2**) and screened out 8 genes to construct a multivariable Cox model, including B3GAT3, EEF1D, NRP1, PLCB1, FASLG, IL2RB, SLC22A7, and STX11, using MMPC algorithm. Notably, expressions of the first four recurrence-related genes and of the remaining ones were negatively and positively correlated with the recurrence-free survival (RFS) in HCC patients, respectively (**Figure 1B**). Consistently, the expressions of upregulated or downregulated genes appeared to be negatively or positively correlated with RFS in HCC, compared with the adjacent normal tissues (**Figure 1C**, Wilcoxon rank sum test, *P* < 0.001). These results indicated that the eight genes were particularly relevant to HCC recurrence.

### Construction and Independent Validation of the Eight-Gene Cox (EGC) Model

**Figure 2:**
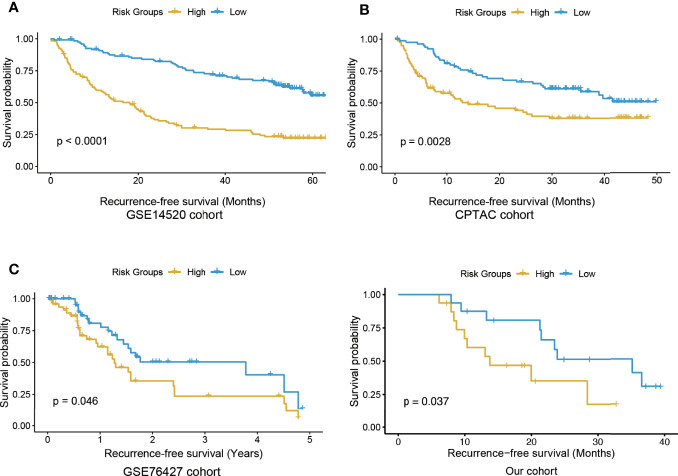
The Kaplan-Meier curves for the high and low risk groups in the training and three validation cohorts. The KM curves of training and three validation cohorts were displayed in **(A–D)**, respectively. The yellow and blue curves represent the high and low risk groups, respectively.

Based on expression profiles of the eight selected genes, we built an Eight-Gene Cox (EGC) model, and HCC patients were stratified into high and low risk groups by the median risk scores (see *Materials and Methods*). Significant differences in RFS between risk groups were observed in the training cohort (GSE14520) (**Figure 2A**, log rank test, P < 0.0001). Consistently, in two validation cohorts [CPTAC (Clinical Proteomic Tumor Analysis Consortium) and GSE76427], the RFS in the high-risk group was shorter (**Figures 2B, C**). Furthermore, we collected 32 snap-frozen HCC tissues, and measured RNA abundance of the eight genes using quantitative real-time PCR (qPCR). Consistently, in this cohort, significantly shorter RFS was observed in the high-risk group, compared with the low-risk group (**Figure 2D** and **Supplementary Table S3**, log-rank test, *P*-value < 0.05), further suggesting that the EGC model could ensure robust predictions of HCC recurrence. In addition, we also compared the performance of MMPC with that of stepwise regression and Lasso algorithms, respectively, and found that MMPC achieved the highest C-index and statistical significance compared with stepwise regression and Lasso algorithms (**Supplementary Table S4**). These results suggested that the EGC model provided an enhanced performance and consistency in HCC recurrence prediction.

### The Risk Stratification by EGC Model Is an Independent Predictor of HCC Recurrence

To further demonstrate that the EGC model was an independent predictor of HCC recurrence, we built a multivariable Cox model based on this risk stratification and four other prognostically relevant risk factors, including Barcelona Clinic Liver Cancer (BCLC) stage, Alpha Fetoprotein (AFP), tumor size, and age. Notably, the validation cohort under accession number GSE76427 was excluded in this analysis, due to a lack of clinical characteristics. In the multivariable Cox model, significant p-values for BCLC and the risk stratification were observed in the training and the validation (CPTAC) cohorts (**Tables 1**, **2**). More importantly, the statistical significance of the risk stratification was higher than BCLC. These results indicated that the risk stratification by EGC model was an independent prognostic factor in HCC recurrence prediction.

**Table 1:** The coefficients and statistical significance of the risk stratification and four prognostic factors in the multivariable model (training cohort).

Factor		Coef.	Exp (coef.)	Se (coef.)	z	Pr (>|z|)	
BCLC							
	A	0.88	2.42	0.43	2.07	3.89E-02	*
	B	1.10	2.99	0.50	2.19	2.84E-02	*
	C	1.67	5.29	0.49	3.41	6.52E-04	*
AFP							
	<300 ng/ml	-0.04	0.96	0.18	-0.24	8.09E-01	
Tumor size							
	<5cm	0.09	1.10	0.21	0.46	6.48E-01	
Age							
	<50 yrs.	-0.20	0.82	0.18	-1.10	2.70E-01	
Risk group							
	Low-risk	-1.15	0.32	0.20	-5.71	1.11E-08	***

*: P-value < 0.05, ***: P-value < 0.001.

**Table 2:** The coefficients and statistical significance of the risk stratification and four prognostic factors in the multivariable model (validate (CPTAC) cohort).

Factor		Coef.	Exp (coef.)	Se (coef.)	z	Pr (>|z|)	
BCLC							
	B	0.09	1.10	0.45	0.21	8.36E-01	
	C	0.89	2.42	0.42	2.12	3.38E-02	*
AFP							
	<300 ng/ml	0.02	1.02	0.24	0.07	9.41E-01	
Tumor size		-0.26	0.77	0.40	-0.64	5.25E-01	
	<5cm						
Age		0.32	1.38	0.24	1.31	1.89E-01	
	<50 yrs.						
Risk group							
	Low-risk	-0.54	0.58	0.24	-2.29	2.19E-02	*

*: P-value < 0.05.

### The EGC Model Outperforms Previously Published Models

**Figure 3:**
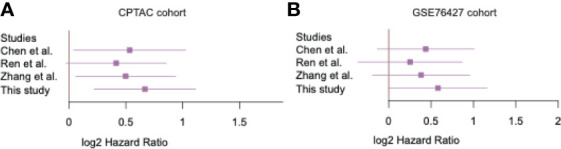
The significance of the recurrence predictive Cox models. The forest plots display the performance of the four Cox models in two validation cohorts **(A, B)**.

To demonstrate superiority of the EGC model, we compared the EGC model with three previously reported HCC recurrence-predicting models. The samples in two validation datasets were also stratified into two risk groups based on expression profiles of different sets of recurrence-related genes from those studies. The comparison of the EGC model and those three models revealed that the EGC model performance was better than the others (9, 30, 31) (**Figures 3A, B**). It should be noted that the other three models were also relatively less significant in GSE76427 cohort. These results demonstrated that the EGC model outperforms previous published models in risk stratification.

### Cell Type-Specific Expression Patterns of the Eight Recurrence-Related Genes

**Figure 4:**
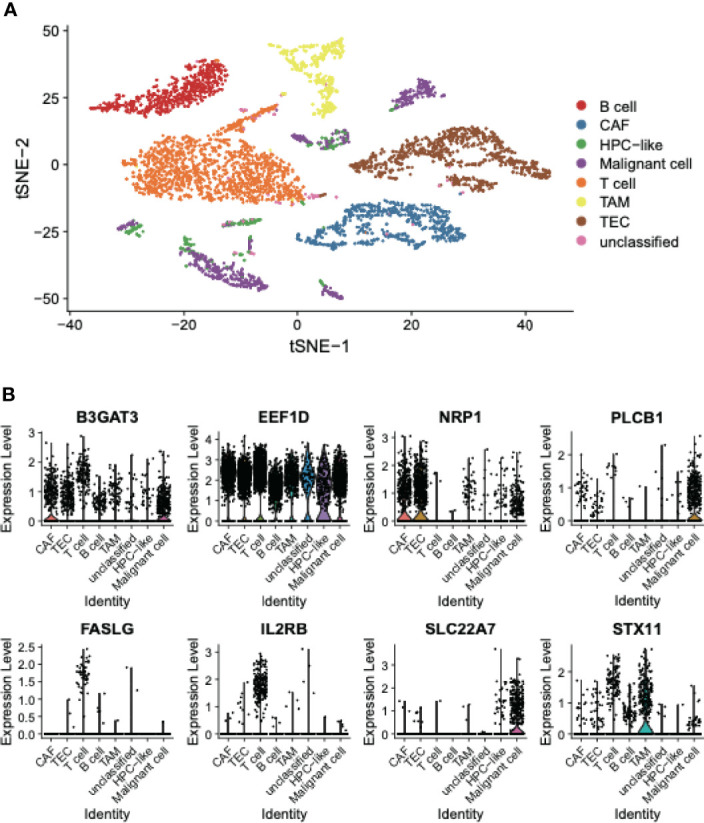
The cell type specific expression in HCC tissues. **(A)** The t-SNE plot represents the distribution of cell types in HCC tissues. **(B)** The expression patterns of the eight recurrence-related genes in the EGC model. The points represent the expression levels of the cells.

To elucidate the expression patterns of these recurrence-related genes in the tumor microenvironment, we collected single-cell RNA sequencing (scRNA-seq) data of 12 HCC samples from a previous study. The t-Distributed Stochastic Neighbor Embedding (t-SNE) analysis and dimensionality reduction of the scRNA-seq data revealed that the cells could be divided into eight cell clusters, and seven out of these cell clusters could be annotated using the cell type specific marker genes reported by Ma L. et al. (21), namely B cells, cancer-associated fibroblasts (CAF), hepatic progenitor cell (HPC)-like, malignant cells, T cell, tumor-associated macrophages (TAM), and tumor-associated endothelial cells (TEC) (**Figure 4A**). Among the eight recurrence-related genes, PLCB1, FASLG, IL2RB, and SLC22A7 were found to be specifically expressed in only one cell type. Specifically, PLCB1 and SLC22A7 were expressed in malignant cells, while both FASLG and IL2RB were expressed in T cells (**Figure 4B**). In contrast, B3GAT3, EEF1D, NRP1, and STX11 were expressed in multiple cell types. These results indicated that the expression patterns of SLC22A7, PLCB1, FASLG, and IL2RB might be cell type-specific.

### Interpretation of Cell-Type Specific Recurrence-Related Genes in HCC

**Figure 5:**
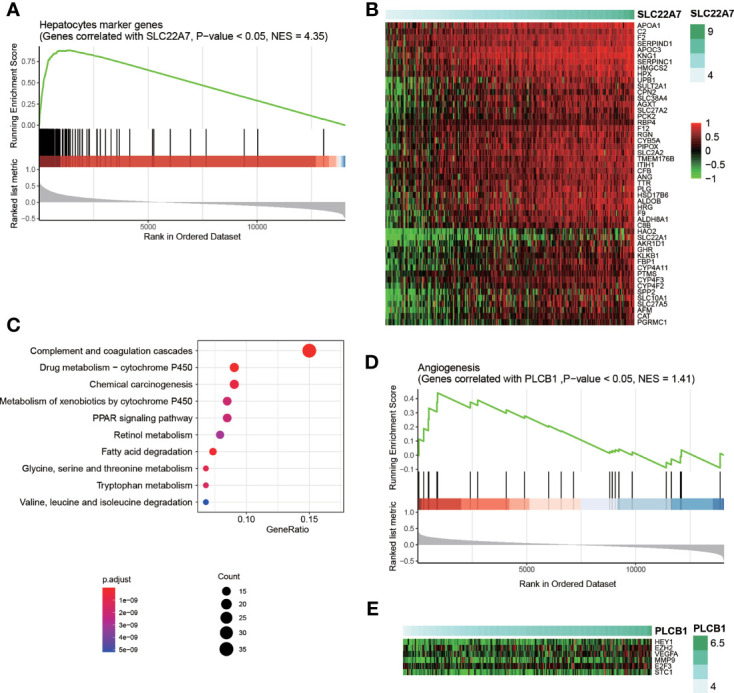
Interpretation of SLC22A7 and PLCB1 in HCC recurrence. **(A)** The statistical significance of the genes correlated with SLC22A7 enriched in the hepatocytes marker genes. The y axis on the bottom represents the Spearman correlation coefficients. **(B)** The heatmap for the core genes positively correlated SLC22A7 and also specifically expressed in hepatocytes. **(C)** The KEGG pathways enriched by the core genes positively correlated SLC22A7 and also specifically expressed in hepatocytes. **(D)** The statistical significance of the genes correlated with PLCB1 enriched in the angiogenesis-related genes. **(E)** The heatmap for the core genes positively correlated PLCB1 and also involved in angiogenesis.

To investigate how those four cell-type-specific genes participated in HCC recurrence, we conducted correlation analysis and gene set enrichment analysis (GSEA) to interpret their functional roles using the training cohort. SLC22A7 was specifically expressed in malignant cells, however, this gene was downregulated in HCC tissues (**Figure 1C**). The genes whose expressions were positively correlated with SLC22A7 expression were highly enriched in the hepatocyte marker genes (**Figure 5A**), further suggesting that SLC22A7 was a hepatocyte-specific marker gene. As shown in **Figure 5B**, highly similar expression patterns of SLC22A7 and of the core genes in those SLC22A7-related ones were observed. The KEGG pathway enrichment analysis of these genes revealed that they were highly enriched in liver-related metabolism (**Figure 5C**), suggesting that SLC22A7 was involved in liver-related metabolism. PLCB1 was also identified as a malignant cell-specific gene, and was upregulated in HCC. The GSEA revealed that the genes correlated with PLCB1, including HEY1, EZH2, VEGFA, E2F3, and STC1, were enriched in angiogenesis (**Figures 5D, E**), suggesting that the increased expression of PLCB1 might be associated with HCC recurrence *via* tumor-associated angiogenesis.

**Figure 6:**
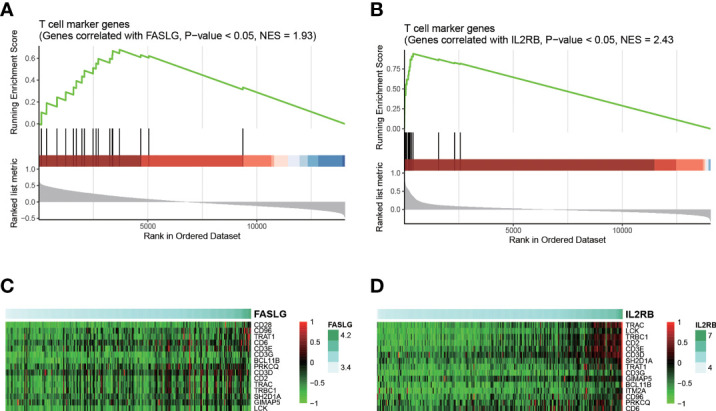
Interpretation of FASLG and IL2RB in HCC recurrence. The statistical significance of the genes correlated with FASLG and IL2RB enriched in T cell marker genes are displayed in **(A, B)**, respectively. The heatmaps for the core genes positively correlated FASLG and IL2RB and also specifically expressed in T cell are displayed in **(C, D)**, respectively.

As FASLG and IL2RB were identified as two candidate T cell specific marker genes, we thus speculated that the downregulation of these two genes might be associated with T cell-mediated anticancer properties. Consistently, expressions of these two genes were positively correlated with expressions of T cell marker genes (**Figures 6A, B**). Notably, CD96, TRAT1, CD6, CD3D, CD3E, CD3G, TRAC, BCL11B, PRKCQ, CD2, TRBC1, SH2D1A, GIMAP5, and LCK were identified as the core genes in T cell marker genes (**Figures 6C, D**). These results indicated that low expression levels of FASLG and IL2RB might be associated with attenuated T cell-mediated anticancer activities, thus resulting in HCC recurrence.

In addition, we also conducted GSEA for the remaining genes, including B3GAT3, EEF1D, NRP1, and STX11, to interpret their biological functions in HCC. Of note, B3GAT3, EEF1D, NRP1, and STX11 were predicted to be involved in gap junction trafficking and regulation, protein translation, collagen formation, and G-protein coupling receptors (GPCRs) signaling according to GSEA, respectively (**Supplementary Table S5**).

### PLCB1 Silencing Decreases the Microvessel Density (MVD) in HCC

**Figure 7:**
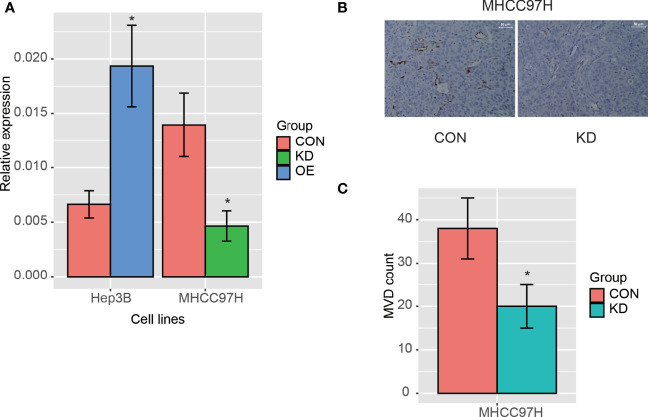
The PLCB1 expression in HCC cell lines. **(A)** The RNA expression of PLCB1 by the knockdown and overexpression of PLCB1 mRNA. **(B)** The microvessel density (MVD) in HCC tissue with PLCB1 knockdown and the control. **(C)** The MVD count in HCC tissue with PLCB1 knockdown and the control. *: P-value < 0.05.

As PLCB1 was upregulated in malignant cells and was predicted to be involved in angiogenesis according to GSEA, we then investigated whether PLCB1 overexpression could lead to increased microvessel density in HCC. Two HCC cell lines, MHCC97H (high metastatic potential) and Hep3B (low metastatic potential), were selected for this analysis, following our previous studies (25, 32). Firstly, we silenced and over-expressed PLCB1 in MHCC97H and Hep3B cells, using the small interface RNA (siRNA) and an PLCB1 expression vector (n = 3), respectively. Nude mice were subcutaneously inoculated with those cells. Then, *in situ* HCC nude mice model was established. As shown in **Figure 7A**, the mRNA expression levels of PLCB1 were significantly reduced after siRNA silencing (KD group), while an increase was observed after PLCB1 overexpression (OE group) (n = 3). Furthermore, we measured the MVD in tumor tissues from the nude mice, and found that the KD group had significantly lower MVD than the controls (CON group) (**Figures 7B, C**, t test, *P*-value < 0.05), indicating that PLCB1 silencing could decrease the MVD in HCC.

### SLC22A7 Inhibits HCC Metastasis by Reducing MMP9 Activity

**Figure 8:**
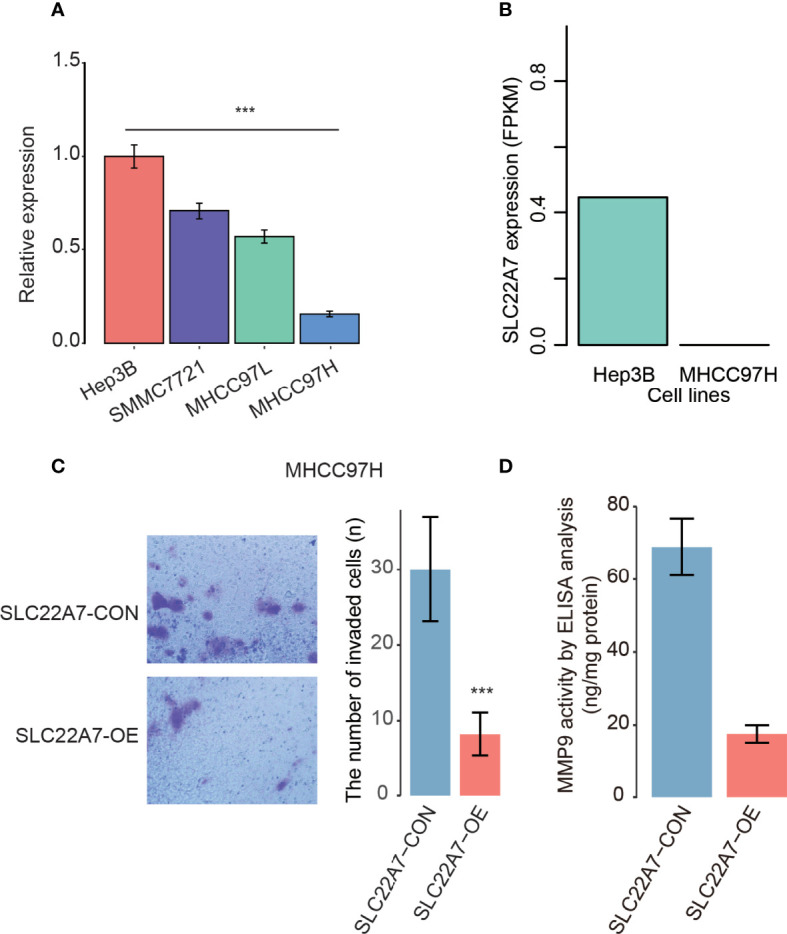
The SLC22A7 expression in HCC cell lines. **(A)** The SLC22A7 expression levels detected by qRT-PCR. The expression levels were normalized to Hep3B. **(B)** The SLC22A7 expression levels quantified by RNA-seq data. **(C)** The number of invaded cells in SLC22A7-overexpression (SLC22A7-OE) and control (SLC22A7-CON) MHCC97H cell lines. **(D)** MMP9 activity in SLC22A7-OE and SLC22A7-CON groups by ELISA analysis. ***: P-value < 0.001.

As SLC227A was specifically expressed in hepatocytes, we then examined its expression patterns in cell lines with varied metastatic potentials. We quantified the expression levels of SLC22A7 in HCC cell lines including Hep3B, SMMC7721, MHCC97L, and MHCC97H. Specifically, we found that SLC22A7 was expressed higher in lowly metastatic Hep3B cells than highly metastatic MHCC97H cells (student-*t* test, *P* < 0.001, **Figure 8A**). Accordingly, lower expression of SLC22A7 in highly metastatic HCC cells was also observed in the RNA sequencing data from a previous study (33) (**Figure 8B**). These results indicated that low expression of SLC22A7 might be associated with HCC metastasis.

To further explore the functional roles of SLC22A7 in HCC metastasis, we overexpressed SLC22A7 in MHCC97H cells, and used MHCC97H cells transfected with the empty vector as controls. Compared with the control group, SLC22A7 overexpression significantly attenuated the invasive ability of MHCC97H cells in Transwell assay (**Figure 8C**). Moreover, we also evaluated the activity of matrix metalloproteinase-9 (MMP9) in MHCC97H using an ELISA kit, and found that MMP9 activity was significantly decreased after SLC22A7 overexpression (**Figure 8D**), suggesting that SLC22A7 might prevent HCC invasion by inhibiting MMP9 activity.

## Discussion

There is still a lack of effective early detection and prognostication methods for HCC, which remains a leading cause of cancer-related mortality worldwide. In the present study, we have attempted to develop a predictive model with optimized markers for HCC recurrence. Specifically, we screened out 8 genes, including B3GAT3, EEF1D, NRP1, PLCB1, FASLG, IL2RB, SLC22A7, and STX11, through a series of data analyses such as differential expression analysis, Cox regression analysis, and MMPC algorithm. The risk stratification based on the Eight-Gene Cox (EGC) model was closely associated with the RFS of HCC patients in both training and three validation cohorts. Of note, the reduced strength of EGC model in the validation cohorts might be resulted from the reduced sample size. We have demonstrated that the EGC model could serve as an independent predictor of HCC recurrence, through a multivariable Cox model constructed with the risk stratification (samples were stratified as either low or high risk) and four prognostically relevant variables. Consistently, the superiority of the EGC model was demonstrated by comparing it with other three models (9, 30, 31). In short, the EGC model could accurately predict the risk of recurrence in HCC.

As single-cell RNA sequencing analysis facilitates the identification of HCC recurrence-related genes and interpretation of their functional roles at a higher resolution, we investigated the expression patterns of selected genes in different cell populations from HCC tissues. Among those eight genes in the EGC model, B3GAT3, EEF1D, NRP1, and STX11 were not expressed in a cell type-specific manner. B3GAT3 is a member of glucuronyltransferase, and is involved in glycosaminoglycans biosynthesis (34). High expression of B3GAT3 has been reported to be associated with poor prognosis in liver cancer (35). Moreover, EEF1D, an eukaryotic translation elongation factor, also exhibits prognostic significance in multiple cancers (36). NRP1, a transmembrane co-receptor for semaphorins and heparin-binding pro-angiogenic cytokines, has been reported to be upregulated in hepatocellular carcinoma, as it contributes to tumor growth and vascular remodeling (37). However, STX11 was found downregulated in HCC tissues, and it has been reported to function as a tumor suppressor gene in peripheral T-cell lymphomas (38). These results suggested that these four genes might also be associated with HCC recurrence in similar manners.

Particularly, PLCB1 and SLC22A7 were predominantly expressed in malignant cells, while both FASLG and IL2RB were specifically expressed in T cells (**Figure 4B**). PLCB1 was identified as a malignant cell-specific genes in HCC, and has been reported to be implicated in breast cancer (39), HCC (40), and ovarian cancer (41). However, its functional role in HCC has not been fully unveiled. In this study, we found that PLCB1 silencing could decrease the MVD in HCC, suggesting that PLCB1 silencing might inhibit the angiogenesis of HCC. SLC22A7, which was downregulated in HCC yet specifically expressed in malignant cells, has been reported to affect mitochondrion and oxidoreductase in noncancerous liver tissues, thereby promoting the occurrence of HCC (42). The KEGG pathway analysis further confirmed that SLC22A7 was associated with normal metabolism in human liver. Kudo et al. reported that SLC22A7 was the best predictor of multicentric occurrence (MO)-free survival (MFS), and SLC22A7 downregulation was associated with mitochondrion and oxidoreductase activity (42). Moreover, reduced SLC22A7 expression in the liver could indicate a significant risk of HCC development in chronic hepatitis C, which was independent of other risk factors as described by Yasui et al. (43). As the hepatocyte-specific markers were abundantly expressed in highly differentiated liver cancer cells, the close association between SLC22A7 and hepatocyte-specific markers suggested that SLC22A7 might participate in tumor differentiation. Moreover, we also observed that SLC22A7 was expressed lower in highly metastatic MHCC97H cells than lowly metastatic Hep3B cells. It is well recognized that histological differentiation grade of HCC is associated with recurrence (44–46). To further explore the functional roles of SLC22A7 in HCC metastasis, we overexpressed SLC22A7 in MHCC97H cells, and found that SLC22A7 overexpression significantly decreased the invasive ability of MHCC97H cells and reduced the activity of matrix metalloproteinase-9 (MMP9), suggesting that SLC22A7 might prevent HCC invasion by inhibiting MMP9 activity. In addition, it is widely reported that FASLG and IL2RB were specifically expressed in T cells (47–50). High infiltration of T cells was associated with low recurrence rate in HCC (51, 52), and cytotoxic T cells were key contributors to anticancer immunity in multiple cancers (53–55). Thus, positive correlation between the expressions of FASLG/IL2RB and T cell markers suggested that FASLG/IL2RB might promote T cell-induced anticancer immunity in HCC.

In addition, the present study still had some limitations. One is the lack of a larger cohort when developing the proposed Cox model. Another limitation is that *in vivo* experiments in HCC mice models should be proposed to validate the molecular mechanisms. In conclusion, the EGC model and eight identified marker genes could not only facilitate the accurate prediction of HCC recurrence, but also improve our understanding of the mechanisms behind HCC recurrence.

## Data Availability Statement

The original contributions presented in the study are included in the article/**Supplementary Material**. Further inquiries can be directed to the corresponding author.

## Author Contributions

X-YaH designed and coordinated the study; Z-LH, BX and T-TL performed the experiments, acquired and analyzed data; X-YaH, Z-LH, Y-HX, T-TL, and X-YuH interpreted the data; Z-LH, BX, and X-YaH wrote the manuscript; all authors approved the final version of the article.

## Funding

This study was supported by grants from the Project funded by the central government to guide local scientific and technological development (YDZX20213100001001), and the Interdisciplinary Program of Shanghai Jiao Tong University (No. YG2017MS13).

## Conflict of Interest

The authors declare that the research was conducted in the absence of any commercial or financial relationships that could be construed as a potential conflict of interest.

The reviewer YY declared a shared parent affiliation with the author YHX to the handling editor at the time of review.

## Publisher’s Note

All claims expressed in this article are solely those of the authors and do not necessarily represent those of their affiliated organizations, or those of the publisher, the editors and the reviewers. Any product that may be evaluated in this article, or claim that may be made by its manufacturer, is not guaranteed or endorsed by the publisher.
