# Supplementary material for: Integrative Analysis Identifies Cell-Type-Specific Genes Within Tumor Microenvironment as Prognostic Indicators in Hepatocellular Carcinoma
Source: Front Oncol. 2022 May 30;12:878923. doi: 10.3389/fonc.2022.878923 (PMC9190278; doi:10.3389/fonc.2022.878923)
Supplement: Supplementary file 4 [file DataSheet_4.docx]

**Supplementary Table S4. The performance of three methods to select features for Cox model construction**

| **Algorithm** | **gene** | **coef** | **exp**  **(coef)** | **se**  **(coef)** | **z** | **Pr(>\|z\|)** | **C-index** | **log-rank test** | **Likelihood ratio test** |
| --- | --- | --- | --- | --- | --- | --- | --- | --- | --- |
| MMPC | B3GAT3 | 0.3979 | 1.4887 | 0.1810 | 2.1981 | 2.7945E-02 | 0.732 | 1.00E-14 | 7.00E-15 |
|  | EEF1D | 0.7153 | 2.0449 | 0.1808 | 3.9557 | 7.6325E-05 |  |  |  |
|  | NRP1 | 0.5240 | 1.6889 | 0.1783 | 2.9398 | 3.2845E-03 |  |  |  |
|  | PLCB1 | 0.5662 | 1.7616 | 0.1825 | 3.1021 | 1.9213E-03 |  |  |  |
|  | FASLG | -0.4009 | 0.6697 | 0.1819 | -2.2042 | 2.7513E-02 |  |  |  |
|  | IL2RB | -0.6588 | 0.5174 | 0.1851 | -3.5591 | 3.7217E-04 |  |  |  |
|  | SLC22A7 | -0.6750 | 0.5091 | 0.1843 | -3.6623 | 2.4999E-04 |  |  |  |
|  | STX11 | -0.3968 | 0.6724 | 0.1783 | -2.2257 | 2.6037E-02 |  |  |  |
| Lasso | AMD1 | 0.0401 | 1.0409 | 0.2645 | 0.1515 | 8.7955E-01 | 0.675 | 0.02 | 0.02 |
|  | CCT2 | 0.1505 | 1.1624 | 0.2860 | 0.5263 | 5.9866E-01 |  |  |  |
|  | CDC25B | 0.5210 | 1.6836 | 0.2732 | 1.9067 | 5.6566E-02 |  |  |  |
|  | GNAZ | 0.4645 | 1.5913 | 0.2502 | 1.8567 | 6.3357E-02 |  |  |  |
|  | MAPK13 | 0.0796 | 1.0829 | 0.2597 | 0.3066 | 7.5912E-01 |  |  |  |
|  | PHLDA2 | 0.0636 | 1.0657 | 0.2499 | 0.2544 | 7.9918E-01 |  |  |  |
|  | PRC1 | 0.1575 | 1.1706 | 0.2506 | 0.6286 | 5.2960E-01 |  |  |  |
|  | RPL8 | 0.1512 | 1.1633 | 0.2512 | 0.6020 | 5.4716E-01 |  |  |  |
|  | SLC7A1 | 0.0239 | 1.0242 | 0.2605 | 0.0919 | 9.2675E-01 |  |  |  |
|  | SSB | 0.2687 | 1.3082 | 0.2867 | 0.9372 | 3.4868E-01 |  |  |  |
|  | TKT | 0.2153 | 1.2402 | 0.2733 | 0.7877 | 4.3085E-01 |  |  |  |
|  | TOMM20 | 0.3887 | 1.4751 | 0.2822 | 1.3777 | 1.6830E-01 |  |  |  |
|  | VIL1 | 0.1214 | 1.1291 | 0.2639 | 0.4601 | 6.4544E-01 |  |  |  |
| stepwise regression | CDC25B | 0.6083 | 1.8374 | 0.2521 | 2.4133 | 1.5810E-02 | 0.652 | 2.00E-04 | 1.00E-04 |
|  | GNAZ | 0.5345 | 1.7065 | 0.2316 | 2.3075 | 2.1027E-02 |  |  |  |
|  | SSB | 0.4814 | 1.6184 | 0.2473 | 1.9469 | 5.1546E-02 |  |  |  |
|  | TOMM20 | 0.5378 | 1.7122 | 0.2654 | 2.0260 | 4.2761E-02 |  |  |  |
